# Effects of novel cellulase (Cel 906) and probiotic yeast fermentation on antioxidant and anti-inflammatory activities of vine tea (*Ampelopsis grossedentata*)

**DOI:** 10.3389/fbioe.2022.1006316

**Published:** 2022-09-15

**Authors:** Jin Xu, Mubasher Hussain, Wenfeng Su, Qian Yao, Guandong Yang, Yu Zhong, Lin Zhou, Xiaoting Huang, Zhixiang Wang, Quliang Gu, Yifei Ren, He Li

**Affiliations:** ^1^ Key Specialty of Clinical Pharmacy, The First Affiliated Hospital of Guangdong Pharmaceutical University, Guangzhou, China; ^2^ Guangdong Key Laboratory of Bioactive Drug Research, Guangzhou, China; ^3^ Guangdong Key Laboratory of Animal Conservation and Resource Utilization, Guangdong Public Laboratory of Wild Animal Conservation and Utilization, Guangdong Engineering Research Center for Mineral Oil Pesticides, Institute of Zoology, Guangdong Academy of Science, Guangzhou, China; ^4^ CAS Testing Technical Services (Guangzhou) Co., Ltd., Guangzhou, China; ^5^ Guangzhou Ruby Biotechnology Co., Ltd., Guangzhou, China; ^6^ Guangdong Molecular Probe and Biomedical Imaging Engineering Technology Research Center, Guangzhou, China; ^7^ Guangzhou Hua Shuo Biotechnology Co., Ltd., Guangzhou, China

**Keywords:** fermentation, vine tea, zebrafish, yeast, antioxidant, anti-inflammatory

## Abstract

Vine tea (*Ampelopsis grossedentata*) is a plant resource with good nutritional and medicinal, and is widely consumed in China. This study aimed to develop a functional vine tea fermentation broth using microbial fermentation and cellulase degradation. First, the most suitable probiotics for vine tea fermentation were screened, and the fermentation conditions were optimized. Then, a new cellulase (Cel 906, MW076177) was added to evaluate the changes in the contents of effective substances and to study its efficacy. The results show that *saccharomyces cerevisiae* Y-401 was identified as the best strain, the optimal fermentation conditions were a time of 94.60 h, feeding concentration of 115.21 g/L, and temperature of about 34.97°C. The vine tea fermentation broth has a strong inhibitory ability on 2,2′-azinobis3-ethylbenzothiazoline-6-sulfonic acid (ABTS) (99.73%), peroxyl (53.15%), superoxide anion radicals (84.13%), and 1,1-Diphenyl-2-trinitrophenylhydrazine (DPPH) (92.48%). It has a decent inhibitory impact on the cell viability, tyrosinase activity (32.25%), and melanin synthesis (63.52%) of B16-F10 melanoma cells induced by α-MSH. Inflammatory cell recruitment was reduced in a zebrafish inflammation model. Therefore, this vine tea fermented broth has strong antioxidant, anti-melanoma, and anti-inflammatory effects, and has healthcare potential as a probiotic tea.

## 1 Introduction

Vine tea, with the scientific name of *Ampelopsis grossedentata* and belonging to Vitiaceae family, is a healthy herb tea rich in natural antioxidant dihydromyricetin (Zhang et al., 2021). Its dried leaves and stems are important plant resources for medicinal food research, and are valued by people because of extremely high nutritional content and medicinal value. They are typically used as medicative and edible plants and are usually consumed by Chinese individuals for prevent of high blood pressure, hyperlipidaemia, non-alcoholic fatty liver disease, for the treatment of veseel and neural structure diseases (Xie et al., 2020). Vine tea contains various active ingredients, such as flavonoids, polyphenols, polysaccharides, thearubigin, selenium, amino acids, and vitamins, these active ingredients are associated with health advantages (Khan et al., 2019; Castaldo et al., 2021; Silvan et al., 2021).

Probiotics are a species of bacteria that provide consumers with health benefits (Soccol et al., 2010). Yeasts, bacilli, *Escherichia coli*, enterococci, and the more commonly-used *bifidobacteria* and lactic acid bacteria, are all probiotic to humans (Soccol et al., 2010). *Saccharomyces boulardii* can forestall enteral infection caused by *Escherichia coli, Salmonella typhimurium, Staphylococcus aureus, Pseudomonas aeruginosa, Proteus vulgaris, Yersinia enterocolitica*, and *Candida albicans* in each *in vitro* and *in vivo* experiments (Czerucka and Rampal., 2002). However, the nutritional function of the probiotic tea of *S. cerevisiae* combined with cellulase and vine tea has not been thoroughly explored.

*S. cerevisiae* has the benefits of short growth cycle, sturdy fermentation ability, simple largescale culture, and wealthy nutrients (e.g., various proteins, sugars, nucleotides, vitamins, minerals and bioactive substances) (Takalloo et al., 2020). It has continually been the most topic of basic analysis and applied research, and is wide utilized in medicine, food and bioethanol production (Sadat and Kodaki, 2014; Ullah et al., 2015; Li et al., 2020).

Fermentation typically refers to varied benign physiological and organic chemistry changes in plants through microorganism metabolism, it could promote the transformation of active ingredients and increase the metabolism of active substances by microorganisms (Zhao et al., 2021). The microbial fermentation technology is widely used in food, medicine, cosmetics, energy (Hashemi et al., 2021; Guo., 2022; Mitri et al., 2022; Zigoski et al., 2021). Recent analysis on vine tea as a fermentation substrate focuses mostly on health care products and other industries, however less on skin effects (Shimizu et al., 2020). In addition, adding cellulase in the microbial fermentation process can damage plant cell walls, promote the precipitation of effective nutrients, and improve efficacy (Xian et al., 2022). The use of the microbial fermentation technology and enzymatic degradation can convert macromolecules in plants into small molecules easily absorbed by the human body and improve the utilization rate of active ingredients (de Souza and Kawaguti., 2021).

In this study, a vine tea fermentation broth was prepared using microbial fermentation and cellulase degradation. Then, the antioxidant ability, anti-inflammatory ability and safety of the vine tea fermented broth were explored, and its nutritional value as a plant functional raw material tea was evaluated.

## 2 Materials and methods

### 2.1 Microorganisms

Yeasts (*S. cerevisiae* Y-8, Y-12, Y-13, Y-15, Y-18, Y- 19, Y-400, Y-401, Y-402, Y-403, Y-501, Y-502, *Debaryomyces hansenii*, *Kluyveromyces marxianus*) was isolated from the mud samples of liquor fermentation cellars in the early stage of the laboratory, *Lactobacillus plantarum*, *Bacillus subtilis*, *Rhododendron*, and *Tremella* were preserved by the Laboratory of Biochemistry and Molecular Biology of Guangdong Pharmaceutical University.

### 2.2 Vine tea and reagent

Vine tea was purchased from Guilin Paradise Lijiang Specialty Products Co., Ltd. (Guangxi Zhuang Autonomous Region, China).

Yeast-extract-powder peptone glucose (YPG) medium, man-rogosa-sharpe (MRS), malt extract powder liquid medium, and LB medium were purchased from Shanghai YSRIBIO Industrial Co., Ltd. (Shanghai, China). Standard flavonoids (rutin, ethanol, sodium nitrite, aluminum nitrate, sodium hydroxide), standard polyphenols (gallic acid, ferrous tartrate, phosphate buffer), and standard polysaccharides (glucose, phenol) were bought from Shanghai Yuanye Bio-Technology Co., Ltd. (Shanghai, China). Dimethyl sulfoxide (DMSO), Roswell Park Memorial Institutefoetal-1640 (RPMI-1640), α-melanocyte-stimulating hormone (α-MSH), fatal bovine serun (FBS), and 3-(4,5-dimethylthiazol-2-yl)-2,5-diphenyltetrazolium bromide (MTT) were synthesized by Procell Life Science & Technology Co., Ltd. (Wuhan, China). 1,1-Diphenyl-2-trinitrophenylhydrazine (DPPH), tris(hydroxymethyl)aminomethane (TRIS), hydrogen peroxide, o-triphenol, ferrous ethylenediaminetetraacetic acid (Fe^2+^-EDTA) solution, methylene blue, hydrogen peroxide, and 2,2′amino-bis (3-ethyl-benzothiazolinesulfonic acid-6) ammonium salt (ABTS) were provided by Tianjin Kemiou Chemical Reagent Co., Ltd. (Tianjin, China).

### 2.3 Strains and media

*D. hansenii*, *S. cerevisiae* in the YPG medium were cultured at 28°C, 120 r/min for 48 h, *K. marxianus* in wort propagation medium was cultured at 30°C, 220 r/min for 24 h, *L. plantarum* in MRS at 37°C, Cultured at 220 r/min for 24 h, *B. subtilis* in Luria-Bertani medium, and *Rhododendron*, *Tremella* in the malt extract powder liquid medium at 37°C, 150 r/min for 24 h, those were cultivated to the logarithmic growth phase with a final concentration of 10^8^ CFU/ml (i.e., OD600 = 1.12) to obtain inoculums (Moon et al., 2020; Park et al., 2020; Li et al., 2021). The strains were placed in sterile glycerin (30% v/v) and keep at −80°C.

### 2.4 Screening most suitable strain for vine tea fermentation

The vine tea was dried in an impact drying oven at 60°C, pulverized by a YB-150 high-speed universal pulverizer (Shanghai Wannian Trading Co., Ltd.), passed through a 60-mesh sieve, and degreased with petroleum ether. Weigh 5 g and sterilization at 121°C and 102.9 kPa on a DSX-24 L instrument (Shanghai Shen’an Medical Instrument Factory) for 20 min, it was stored for later use.

The initial fermentation system of vine tea consisted of sterile vine tea powder, sterile water and strain inoculums. A fermentation system without any strain was set as the control group, and the strains were added for fermentation as the experimental groups. Fermentation was carried out under the optimum conditions (Zhou et al., 2020; Chen et al., 2021), clearance rate, flavonoid contents, polyphenol contents, and polysaccharide contents of each group of fermentation broths were measured, and then the most suitable strains for vine tea fermentation were preliminary determined.

#### 2.4.1 Detection of DPPH clearance rate

The antioxidant capacity of the fermentation broth was preliminarily detected by using the DPPH clearance rate according to the method of Chen et al. (2021). DPPH radical has the maximum absorption peak near 517 nm. When DPPH radical reacts with antioxidant, the absorption value at 517 nm wavelength decreases, and the degree of decrease is quantitatively related to the antioxidant activity of scavenging free radicals.

#### 2.4.2 Establish standard curve and detect active substance content

A standard curve of rutin with absorbance as the ordinate and rutin concentration (0–2.0 mg/ml) as the abscissa was drawn and measured using the enzyme label analyzer (HBS-1096A), the contents of flavonoids in the fermentation broths were measured according to the method of Liu et al. (2021). And draw the gallic acid standard curve and the glucose standard curve in a similar way to measure the content of polyphenols and polysaccharides in the fermentation broth (Galvão et al., 2014; Xie et al., 2019).

### 2.5 Optimization of fermentation conditions

#### 2.5.1 Single-factor analysis of effects of fermentation conditions on antioxidant activity

Seven single-factor conditions were set up to analyze vine tea fermentation conditions, including fermentation time (h), fermentation temperature (°C), strain inoculum (relative to total volume of the fermentation system, %), vine tea concentration (g/L), glucose dosage and nitrogen source dosage (both relative to feed weight, %), and pH. The optimal conditions were screened by the DPPH scavenging rate in the fermentation broths.

#### 2.5.2 Plackett-Burman design

The Plackett-Burman design was used to investigate the effects of the seven single factors above on the antioxidant activity of the fermentation broths under different conditions. In brief, the DPPH scavenging rate was used as the response value based on single-factor analysis. As a result, the high level (+1) and low level (+1) of each factor were taken to determine the significant influencing factors, and each group of experiments was repeated 3 times.

#### 2.5.3 Box-behnken design

Three factors that significantly affected the antioxidant activity were obtained through factorial analysis, and a response surface optimization experiment with N = 17 and DPPH scavenging rate as the response value was designed on Design-Expert 10.0. The experimental results were statistically analyzed to study the effect of the three-factor interaction on DPPH clearance rate, and the optimal fermentation conditions were determined. Each group of experiments was performed in triplicate.

### 2.6 New cellulase Cel 906 added for extraction of effective substances

The new cellulase, Cel 906, was screened within the microbic metagenomic library made by our team within the early stage of the experiment using the soil of the asafoetida subdivision (45° 40′ N, 85° 30′ E) in the Junggar Basin, Xinjiang as a sample. The gene sequence number was submitted to the National Center for Biotechnology Information Database (NCBI) under the gene accession number of MW076177, and its sequence can be searched and downloaded. The enzymatic properties of the new cellulase are optimum temperature at 35°C and optimum pH 7, where it has high resistance against organic solvents and detergents. The optimum addition amount was selected according to the influence of different addition amounts of Cel 906 on the DPPH clearance rate of fermentation broths.

The total flavonoids and polyphenols of vine tea were extracted by a heat reflux method at 52°C and with 72% ethanol for 43 min (Zhang and Lee., 2021), and the extraction was performed 3 times. The total polysaccharides of vine tea were extracted first by ultrasonic-assisted extraction at 480 W power for 16 min and then by heat reflux extraction for 30 min (Hu et al., 2022), and the extraction was also performed 3 times. According to Sections 2.4.2, the flavonoid, polyphenol, and polysaccharide contents in the extracts of the vine tea fermentation broth were measured as C, and the quality of vine tea added in the fermentation system was Q. Then the extraction rate of various effective substances in the fermentation broth was calculated as:

$$Extraction\;rate=\frac{C}{Q}\times 100\%$$ (1)

### 2.7 Antioxidant activity of vine tea fermentation broth *in vitro*

The *in vitro* antioxidant experiments of the vine tea fermentation broth included the measurement of DPPH, superoxide anion, hydroxyl radical and ABTS clearance abilities.

The DPPH clearing ability was measured the same as in Section 2.4.1. The superoxide anion and hydroxyl radical scavenging abilities were monitored by the pyrogallol method and the methylene blue (MB) method, respectively. The ABTS free radical clearance ability was detected using the ABTS assay (Hu et al., 2021; Zheng et al., 2021).

### 2.8 Inhibition experiment of vine tea fermented broth on melanin production

#### 2.8.1 Cell culture

Mouse melanoma cell line B16-F10 (CL-0319) was purchased from Wuhan Procell Life Technology Co., Ltd. (Procell). Cells were genteel in RPMI-1640 medium supplemented with 10% FBS and 1% streptomycin (100 μg/ml)/penicillin (100 units/ml) at 37°C in 5% CO_2_.

#### 2.8.2 Screening suitable fermentation broth concentration

Nine groups were set up: a control group (A0), six groups treated with RPMI-1640 medium and α-MSH (100 nM) at concentrations of 20, 10, 5, 2.5, 1.25, 0.625% (Ax), a negative control group treated with only α-MSH (A01), and a positive control group treated with 500 ug/mL kojic acid and α-MSH (Axo).

The appropriate fermentation broth concentration was screened using the MTT method (Lee et al., 2021). Cell viability inhibition rate was computed as:

$$Cell\;viability=\frac{1-\left(Ax-A0\right)}{A01-A0}\times 100\%$$ (2)

#### 2.8.3 Intracellular tyrosinase activity assay

Six groups were established: a control group (A0), the negative control group was treated with only α-MSH (100 nM) (A01), a positive control group treated with α-MSH (100 nM) and 500 ug/mL kojic acid (Axo), and three experimental groups treated with vine tea fermentation broths at three concentrations and α-MSH (100 nM) (Ax).

Dopa rate oxidation method was used to detect the inhibition rate of tyrosinase activity in B16-F10 melanoma cells (Huang et al., 2014):

$$Inhibition\;rate=\frac{1-\left(Ax-A0\right)}{A01-A0}\times 100\%$$ (3)

#### 2.8.4 Melanin synthesis inhibition test

Groups were established the same as in Section 2.8.3. The inhibition rate of melanin synthesis in B16-F10 melanoma cells was detected by sodium hydroxide lysis. A density of (4–5) ×10^5^ cells/mL was applied to 6-well plates to seed B16 melanoma cells, with 2 ml per well. After culturing for 1 day in a 5% CO_2_ incubator at 37°C, the supernatant was discarded, and 2 ml was supplementing to every well of each group to continue culturing for 24 h. Then after digestion with trypsin, the resulting mixture was collected in a 1.5 ml centrifuge tube and centrifuged at 1,000 rpm for 5 min. After discarding the supernatant, 300 µl of a 1 mol/L NaOH solution containing 10% DMSO was added. The system was moved to a water shower at 80°C for 30 min. Then the melanin-dissolved solution (100 µl in each well) was added to a 96-well plate, with three duplicate wells. The absorbance was measured at 490 nm. The experiment was repeated 3 times. The formula for the inhibition rate of melanin synthesis is the same as that in 2.8.3.

### 2.9 Anti-inflammatory effect experiment

#### 2.9.1 Collection of fishes and fertilized eggs

The transgenic zebrafish Tg (corola: EGFP) used here was introduced by Guangdong Pharmaceutical University (Guangzhou, China) and expanded in our laboratory. Sexually mature zebrafishes were reared in separate tanks between males and females in zebrafish culture units at water temperature 26 ± 2°C, pH 7.2, conductivity 520 μs/cm, and light/dark cycle 14 h:10 h. One day before the experiment, the males and females were paired at the ratio of 1:2, and the eggs were naturally mated.

#### 2.9.2 Macrophage and neutrophil aggregation assay

Tg (corola: EGFP) transgenic zebrafish juveniles at normal three dpf (days post fertilization) were selected, pre-treated with a 0.625, 1.25 or 2.5% vine tea fermentation broth for 1 h, and cultured in water. For the Control group, 50% of the caudal fins of zebrafish larvae were excised with a scalpel under a stereo microscope, and placed in a 6-well cell culture plate (14 cells/well). The wells were added with different concentrations of a vine tea fermentation broth, and further incubated in the incubator for 6 h. After that, the zebrafish larvae were anesthetized with 0.02% tricaine, and the aggregation of macrophages and neutrophils within the tail fin wound of the larvae was determined underneath a fluorescence microscope, and therefore the range of cells was counted (Elks et al., 2011). After the experiment, the zebrafish embryos used in the experiment were inactivated in 2–8°C ice water, and disposed as general waste.

### 2.10 Chick embryo chorioallantoic membrane test for eye irritation/corrosion of vine tea fermentation broth

#### 2.10.1 Chicken embryos

Fertilized embryos of white laghorn chicken after purchase from Guangdong Xinxing Dahuanong Poultry & Egg Co., Ltd. (SPF grade) (Guangzhou, China) were placed in a Rcom MARU MAX incubator with the air chamber side up at 37.6 ± 0.1°C and relative humidity of 46 ± 1%. The eggs were automatically turned once per hour with a horizontal tilt of 45°, and automatically ventilated.

#### 2.10.2 Eye irritant/corrosive hens egg test chorioallantoic membrane

The chicken embryos cultured to 9 days of age were inspected by egg inspection, and the air chamber was opened. A 0.9% NaCl solution, a 0.1 mol/L NaOH solution, and a fatty alcohol ether sulfate sodium salt mixture (Texapon ASV: sodium magnesium laury-myristyl-6- ethoxy-sulphate) were used in the negative control group, the positive control group, and the standard positive control group respectively. In the experimental groups, vine tea fermentation broths with concentrations of 0.625, 1.25 and 2.5% were selected. The experiments were repeated 6 times.

During end point scoring (Budai et al., 2021), the bleeding, coagulation and vascular dissolution were observed after exposure to CAM for 3 min in each group, and the endpoint score (ES) was calculated. The average value of the mathematical sum of the chicken embryo scores, according to the ES, was calculated to classify the eye irritation of the test objects (Supplementary Table S1).

### 2.11 Statistical analysis

All experimental results are expressed as mean ± standard deviation. All data were analyzed on Excel 2010 (Microsoft, Washington, DC, United States) and GraphPad Prism 6 (GraphPad Software, Inc, San Diego, CA, United States). Statistical comparisons between groups were performed using one-way analysis of variance (ANOVA) followed by Tukey’s posttest for multiple comparisons. Differences were considered significant at *p* < 0.05 and below.

## 3 Results and discussion

### 3.1 Screening most suitable strains for vine tea fermentation

#### 3.1.1 Detection of DPPH clearance rate

**FIGURE 1:**
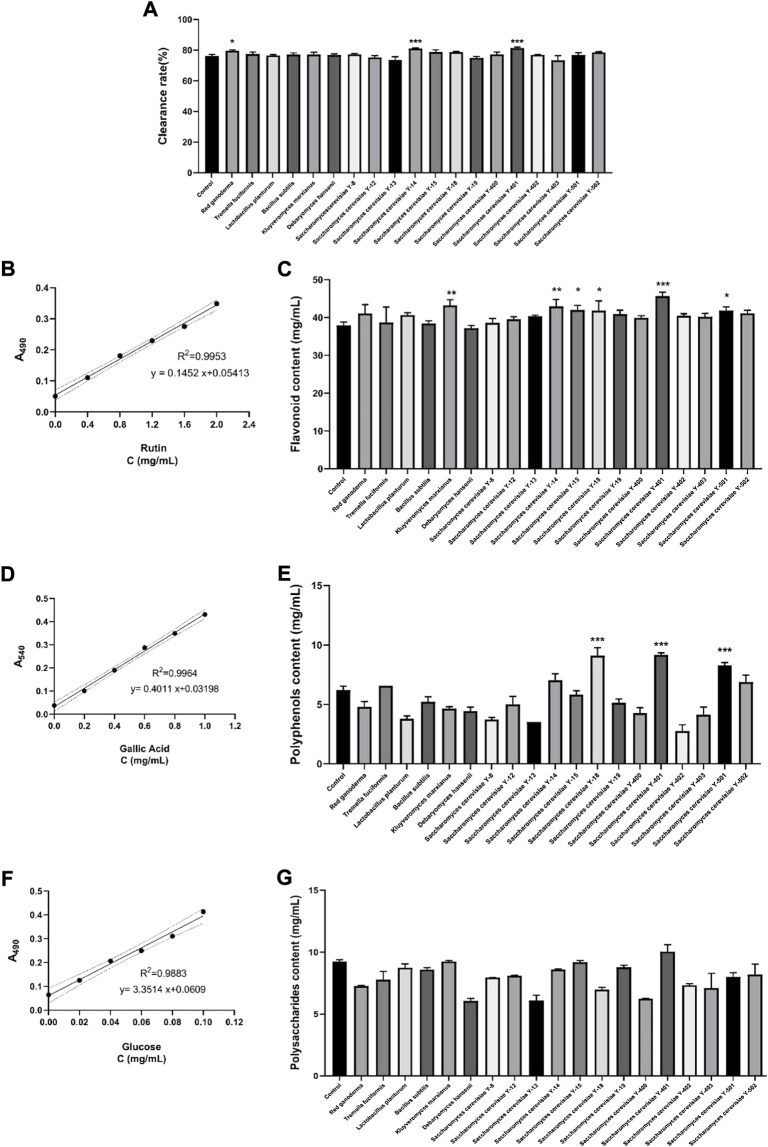
Screening most suitable strains for vine tea fermentation. **(A)** DPPH clearance rate, **(B)** Standard curve of rutin (within 95% CI) **(C)** Flavonoid content, **(D)** Standard curve of gallic acid (within 95% CI) **(E)** Polyphenol content, **(F)** standard curve of glucose (within 95%CI) **(G)** Polysaccharide content. (*p < 0.05, **p < 0.01, ***p < 0.001 versus Control group).

The DPPH clearance rates of the vine tea fermentation broths of different strains were detected (Figure 1A). The fermentation broth of *S. cerevisiae* Y-401 was found to have the significantly highest DPPH scavenging rate (81.36%) (*p* < 0.001). The enzymes secreted by *S. cerevisiae* during the fermentation process release more active ingredients in plants, increase the antioxidant capacity, and increase the DPPH free radical scavenging rate (Ramita et al., 2022).

#### 3.1.2 Standard curve of rutin and content of flavonoids

The standard curve drawn according to different concentrations of rutin is expressed as y = 0.1452 x+0.05413 (R2 = 0.9953), within 95% confidence interval (CI) (Figure 1B). According to the standard curve, the content of flavonoids in the vine teain fermentation broths of each group was calculated. Compared with the control group (Figure 1C), the flavonoid content of the Y-401 group was significantly the highest (45.67 mg/ml) (*p* < 0.001). The enzymes secreted during the growth of *S. cerevisiae* promote the production of flavonoids, and *S. cerevisiae* can also be used as a host for the production of plant flavonoids to increase the content of flavonoids in the fermentation broth (Shota et al., 2022).

#### 3.1.3 Standard curve of gallic acid and content of polyphenols

The standard curve drawn according to different concentrations of gallic acid is y = 0.4011 x + 0.03198 (*R* ^2^ = 0.9964), within 95%CI (Figure 1D). According to the standard curve, the polyphenol content in the fermented vine tea of each group was calculated. Compared with the control group (Figure 1E), the Y-401 group had significantly the highest polyphenol content (9.17 mg/ml) (*p* < 0.001). Cellulases secreted by *S. cerevisiae*, pectinases help plants release bound and free polyphenols, increasing polyphenols in fermentation products (Xu et al., 2022).

#### 3.1.4 Glucose standard curve and polysaccharide content

The standard curve drawn according to different concentrations of glucose is y = 3.3514 x + 0.0609 (R2 = 0.9883), within 95%CI (Figure 1F). According to the standard curve, the polysaccharide content in each group of vine tea fermentation broth was calculated. Compared with the control group, the polysaccharide content of each group decreased (Figure 1G), which may be related to the sugar conversion in fermentation broth into alcohol and CO_2_ by *S. cerevisiae*. Other strains may consume sugar during growth fermentation (Parapouli et al., 2020).

Finding the most suitable strain is the core step of the vine tea fermentation experiments, and determines the change of the effective substance content of the fermentation broth. According to the measured DPPH clearance rate, flavonoid content, polyphenol content and polysaccharide content in each group of fermentation broth, *S. cerevisiae* Y-401 was finally selected as the fermentation strain of vine tea. Since *S. cerevisiae* can secrete various enzymes, such as cellulase, xylanase and pectinase, these enzymes have strong decomposition ability, which can effectively reduce the cell wall obstruction against the extraction of active ingredients, thereby improving the utilization rate. In addition, *S. cerevisiae* can structurally transform compounds through reactions such as methylation, demethylation, dehydroxylation, hydroxylation, deglycosylation, glycosylation, dehydrogenation and hydrogenation, making effective substance content increase or new substances appear (ul Hassan et al., 2020).

The flavonoids, polyphenols and polysaccharides contained in vine tea are active substances that are very beneficial to the human body. They can help people to resist oxidation and inflammation, and have high nutritional value (Carneiro et al., 2021).

### 3.2 Optimization of fermentation conditions

#### 3.2.1 Single-factor analysis of effects of fermentation conditions on antioxidant activity

**FIGURE 2:**
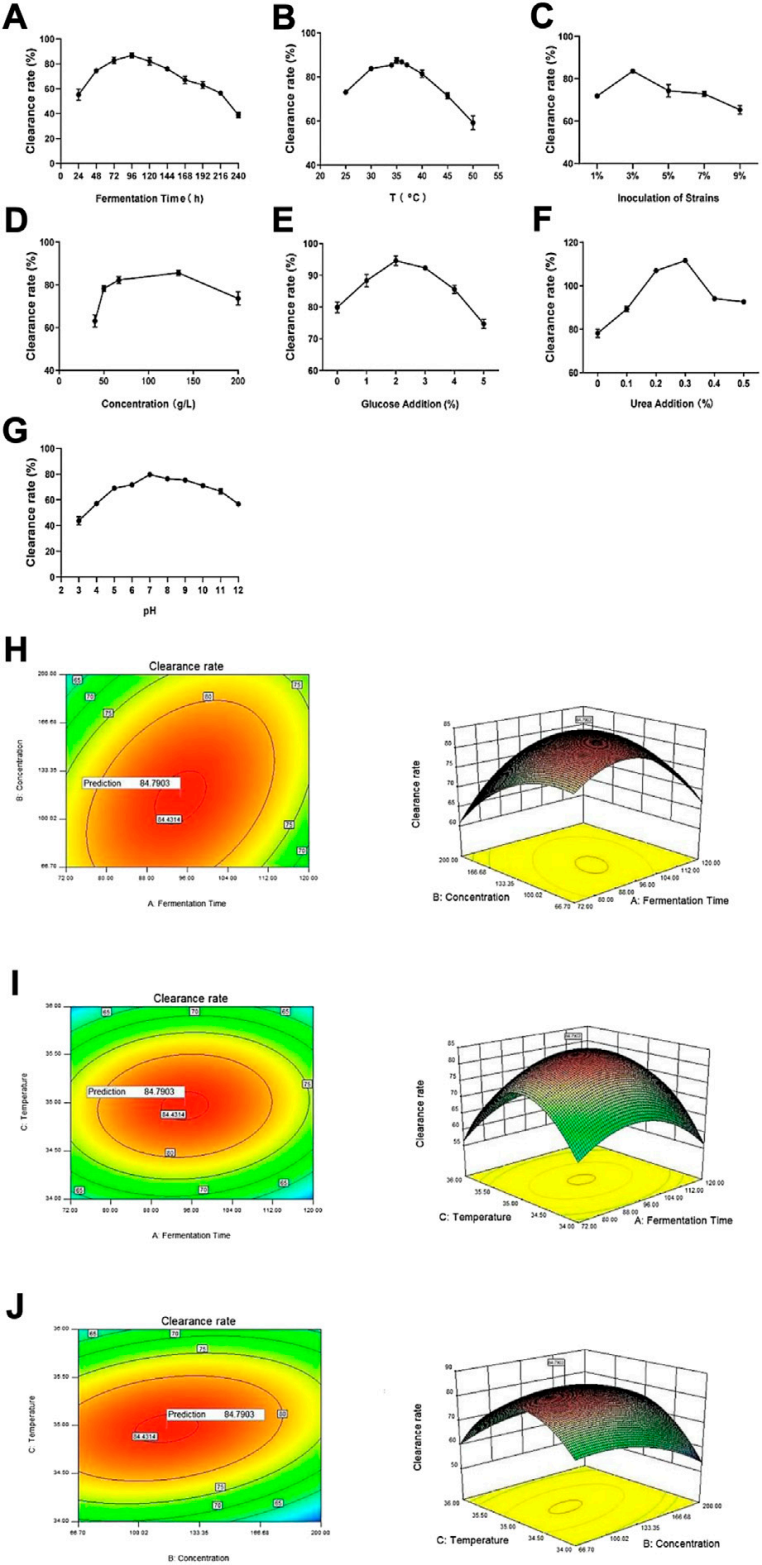
Optimization of fermentation conditions. **(A)** Fermentation time, **(B)** temperature **(C)** strain inoculation amount, **(D)** concentration **(E)** glucose addition, **(F)** urea addition **(G)** pH. And response surface and contour plots of the interactive effects of **(H)** fermentation time and concentration **(I)** fermentation time and temperature and **(J)** temperature and concentration.

To explore the effects of different fermentation conditions on the vine tea fermentation broths, we adopted seven experimental procedures in the single-factor experiment, and determined the optimal fermentation conditions according to the DPPH clearance rates of the fermentation broth under different conditions. The clearance rate increased gradually with the extension of fermentation time from 24 to 96 h, maximizing at 96 h, and slowly decreased from 96 to 240 h (Figure 2A). Under the effects of fermentation temperature, strain inoculum, vine tea concentration, glucose addition, urea addition and pH, the DPPH clearance rate showed a similar trend (Figures 2B–F). The DPPH clearance rate maximized when the fermentation temperature was 35°C, the inoculum amount of the strain was 3%, the concentration was 133.35 g/L, the glucose dosage was 2%, and the amount of nitrogen source was 0.3%, when pH was 7. Appropriate fermentation conditions favor the growth and activity of microorganisms and thereby promote the release and hydrolysis of effective substances, resulting in different antioxidant properties (Takeyama et al., 2022). However, excessively high temperatures can lead to microorganism inactivation, which may affect the fermentation, and excessively long fermentation time can cause loss and consumption of effective substances. The addition of glucose and urea provides carbon and nitrogen sources for the growth of microorganisms, but excessive amounts will inhibit the utilization of fermentation substrates by microorganisms, resulting in reduced release of active substances from vine tea tissues. An appropriate addition of microorganisms will promote the precipitation of effective substances, but addition of too many strains will lead to growth inhibition, which is nonconducive to growth and cannot effectively help the precipitation of active substances in rattan tea (Liu et al., 2021).

#### 3.2.2 Plackett-Burman design results

Plackett-Burman design can analyze the factors that significantly affect the fermented broth of vine tea. Herein, seven factors that affect the antioxidant capacity of vine tea fermented broth were analyzed, and the DPPH clearance rate was the response value. The Plackett-Burman design was completed on Design-Expert 10.0, and the results are shown in Supplementary Material.

The multivariate linear model of the seven factors and the DPPH clearance rate of vine tea fermentation broths was obtained by Plackett-Burman factorial analysis: R1 = +61.57 + 10.19A + 3.69B + 0.34C + 9.56D-0.40E-0.52F-2.45G, where R1 is the DPPH clearance. Analysis of variance shows that the determination coefficient of the model is R2 = 0.9760, indicating that 97.60% of the variability of the experimental data can be explained by this regression equation. The *p* value in the model is 0.0044, indicating the model is significant within 95%CI. A smaller *p* value means the factor has more significant effect on the DPPH clearance rate of the fermentation broth within 95%CI. The order of the influence among the seven factors on the DPPH clearance rate of fermentation broths is fermentation time > vine tea concentration > fermentation temperature > pH > urea addition > glucose addition > strain inoculum. Hence, the three most significant influence factors on the DPPH clearance rate of fermentation broths are fermentation time (*p* = 0.0009), concentration (*p* = 0.0011), and temperature (*p* = 0.0327) (Ko et al., 2022).

#### 3.2.3 Box-Behnken design results

The Box-Behnken design was completed on Design-Expert 10.0, and the DPPH clearance rate of vine tea fermentation broths was the response value. The level design of the three factors (fermentation time, concentration, temperature) and the experimental results was shown in Supplementary Tables S5, S6. Regression fitting of the experimental data yields the following model: R1 = +84.46 + 0.48A-2.57B + 0.38C + 5.51AB+2.03AC+4.75BC-9.37A2-5.58B2-16.98C2, where R1 is the DPPH clearance rate, A, B and C refer to the fermentation time, vine tea concentration and the fermentation temperature respectively. The coefficient of determination R2 of this model is 0.9824, and the adjusted R2 (AdjR2) is 0.9655, indicating this model fits the actual situation. With an accuracy of 98.24%, it can be used to analyze and predict the fermentation conditions of rattan tea. As for the interaction terms, the *p* values of AB, BC, and AC are all greater than 0.05, indicating that there is no interaction within each pair of factors.

The response surface curve and contour map of fermentation time, concentration and temperature on the DPPH clearance rate of vine tea fermentation broths were drawn on Design-Expert.10. The optimal level of the model was predicted through software analysis. Clearly, the best optimized value of each factor is fermentation time of 94.60 h, feeding concentration of 115.21 g/L, and temperature at 34.97°C (Figures 2H–J). Under this condition, experimental verification was carried out, and the average of three parallel experiments was calculated. The DPPH clearance rate of the fermentation broth is 85.4812%, which is close to the predicted maximum clearance rate of 84.7903%, indicating the model is credible.

### 3.3 New cellulase Cel 906 added to help extraction of effective substances

**FIGURE 3:**
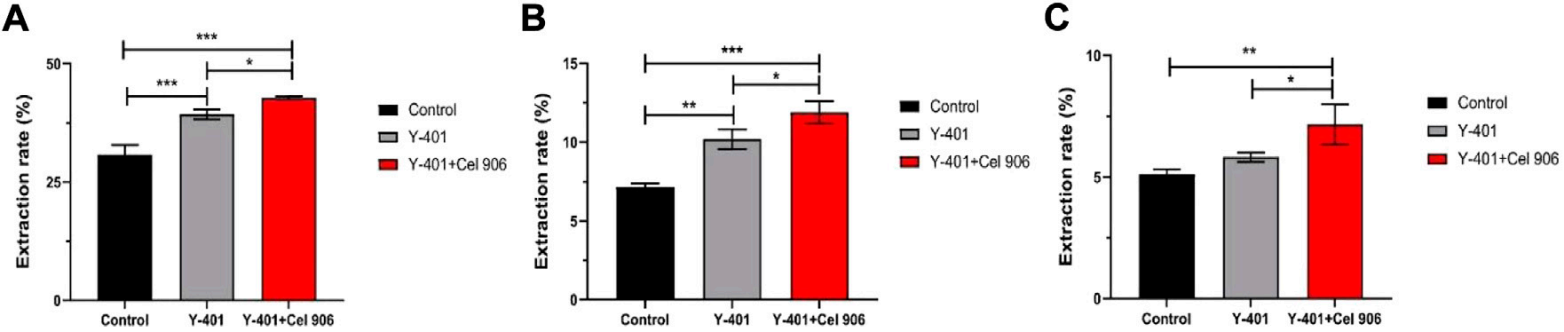
Extraction rate of effective substances. **(A)** Flavonoids, **(B)** polyphenols and **(C)** polysaccharides. (*p < 0.05, **p < 0.01, ***p < 0.001)

Cellulase can hydrolyze the walls of plant cells, increase the extraction rate of effective substances in plant cells and improve the utilization rate (Rostami, and Gharibzahedi, 2017; Zhao et al., 2021). As a result, more effective substances enter the fermentation system and can convert macromolecular substances into small-molecular substances that can be easily absorbed by the skin (Sun et al., 2020). We chose the addition amount of Cel 906 to be 4% (relative to the volume of the fermentation broth). The results of the effect of the addition amount of Cel 906 on the antioxidant activity of the fermentation broth are shown in the Supplementary Figure S1. In the fermentation process of *S. cerevisiae* Y-401, the addition of Cel 906 greatly accelerated the extraction rates of flavonoids (42.79%) and polyphenols (11.90%) (Figures 3A,B). Surprisingly, compared to the control group and the Y-401 group, the vine tea fermentation broth added with Cel 906 significantly increased the polysaccharide extraction rate (*p* < 0.05) up to 7.16% (Figure 3C). Therefore, the treatment with *S. cerevisiae* Y-401 and Cel 906 is very helpful for the extraction and precipitation of vine tea effective substances.

### 3.4 External antioxidant activity of vine tea fermented broth

**FIGURE 4:**
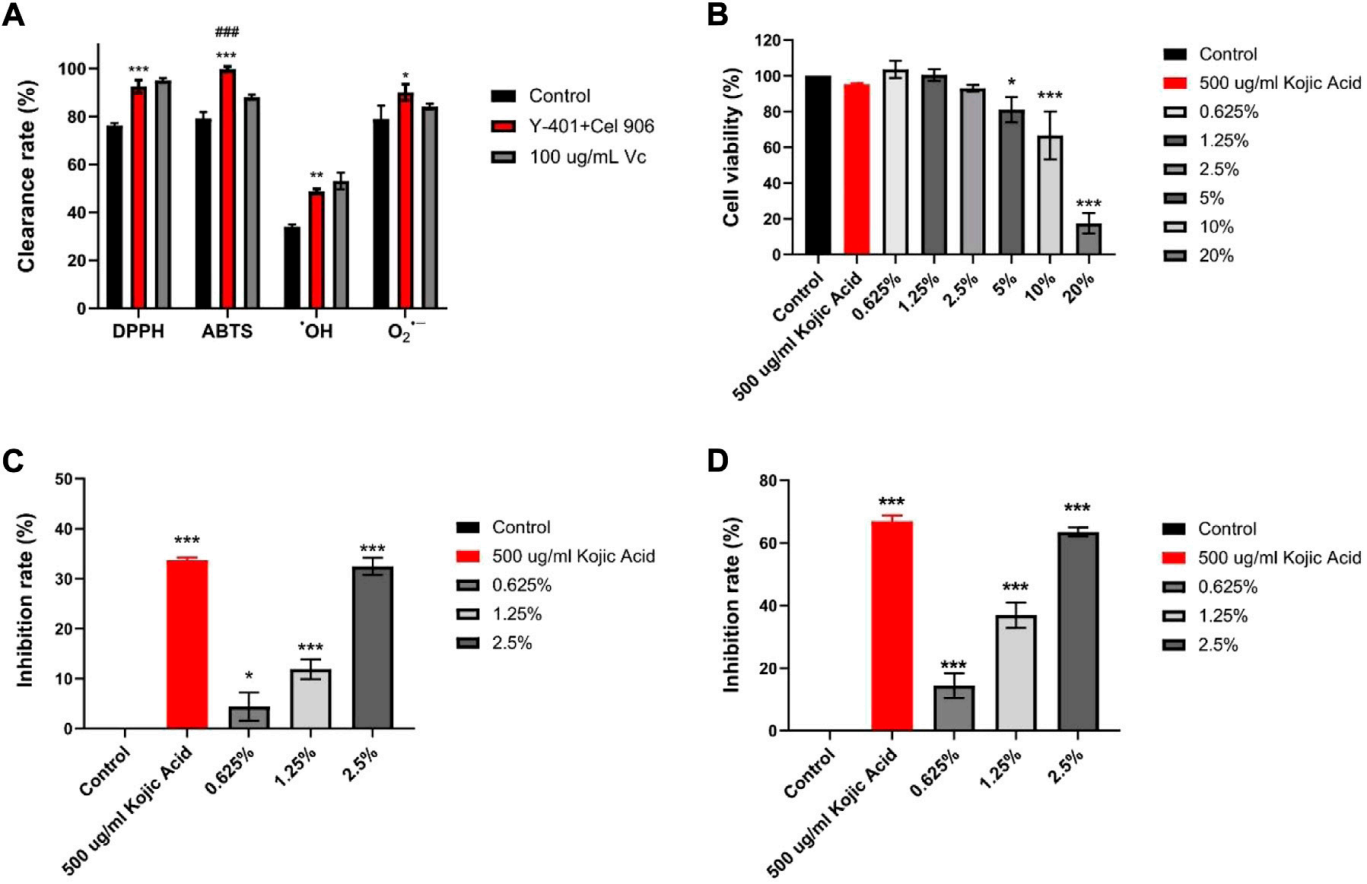
Inhibition of melanin production experiments. **(A)** Statistical chart of DPPH, ABTS, OH-, O2- free radical scavenging ability, **(B)** B16-F10 melanoma cell viability **(C)** Tyrosinase inhibition rate, **(D)** melanin synthesis inhibition rate (*p < 0.05, **p < 0.01, ***p < 0.001 vs Control group. ###*p* < 0.001 vs 100 ug/mL Vc group).

Four methods were used to detect the scavenging capacities of DPPH, ABTS, •OH, and O2•− free radicals respectively in the *in vitro* antioxidant experiments of vine tea fermentation broths. Compared with the control group, the vine tea fermentation broth of the Y-401 and Cel 906 group significantly increased the clearance rate (Figure 4A). Noticeably, the scavenging ability of ABTS free radical (99.73%) of the Y-401 and Cel 906 groups was higher than that of 100 µg/ml Vc (88.04%). Hence, the vine tea fermentation broth treated with *S. cerevisiae* Y-401 and Cel 906 had strong antioxidant activity. Microbial fermentation and cellulase can enhance the antioxidant activity of vine tea fermentation broth, which may promote the rupture of plant cells and the outflow of intracellular antioxidant substances, for example polyphenols, polysaccharide and flavonoids (Wojdyło and Nowicka, 2021; Ji, Xiaolong et al., 2022). Moreover, microorganisms may produce antioxidant factors during fermentation (Zhong et al., 2021). The fermented broth of vine tea can be utilized by the human body as a natural antioxidant.

### 3.5 Inhibition experiment of vine tea fermented broth on melanin production

#### 3.5.1 Screening suitable drug concentrations by MTT

Compared with the control group, the fermentation broths at the concentration of 5%–20% significantly differed in the inhibition of mouse melanoma B16-F10 cell viability (Figure 4B). The 0.625%–2.5% fermentation broth had no significant effect on cell viability. Therefore, three concentrations of 0.625, 1.25, and 2.5% were selected for the subsequent experiments with good biocompatibility (Martyasari and Prasedya, 2021). The vine tea fermented broth has inhibitory effect on the viability of mouse melanoma B16-F10 cells, and the inhibitory effect is stronger as the concentration increases.

#### 3.5.2 Intracellular tyrosinase inhibition

With the inhibition rate of melanin synthesis in the control group being 0, the inhibition rate of tyrosinase gradually increased with the rise of the fermentation broth concentration. Compared with the control group (Figure 4C), the tyrosinase inhibition rates of the fermentation broth at three experimental concentrations were significantly different, and the tyrosinase inhibition rate of the 2.5% fermentation broth (32.45%) was similar to that of kojic acid (33.67%). Tyrosinase is a pivotal enzyme in melanin synthesis, so inhibition of tyrosinase activity can reduce melanin production (Gam et al., 2021).

#### 3.5.3 Melanin synthesis inhibition rate

With the melanin synthesis inhibition rate in the Control group as 0, the melanin synthesis inhibition rate gradually increased with the rise in the concentration of the fermentation broth. The result at the concentration of 2.5% (63.52%) was similar to that of 500 ug/ml kojic acid (66.93%) (Figure 4D). This indicates that the fermented broth of rattan tea has the potential as functional food.

### 3.6 Anti-inflammatory effect of vine tea fermented broth

**FIGURE 5:**
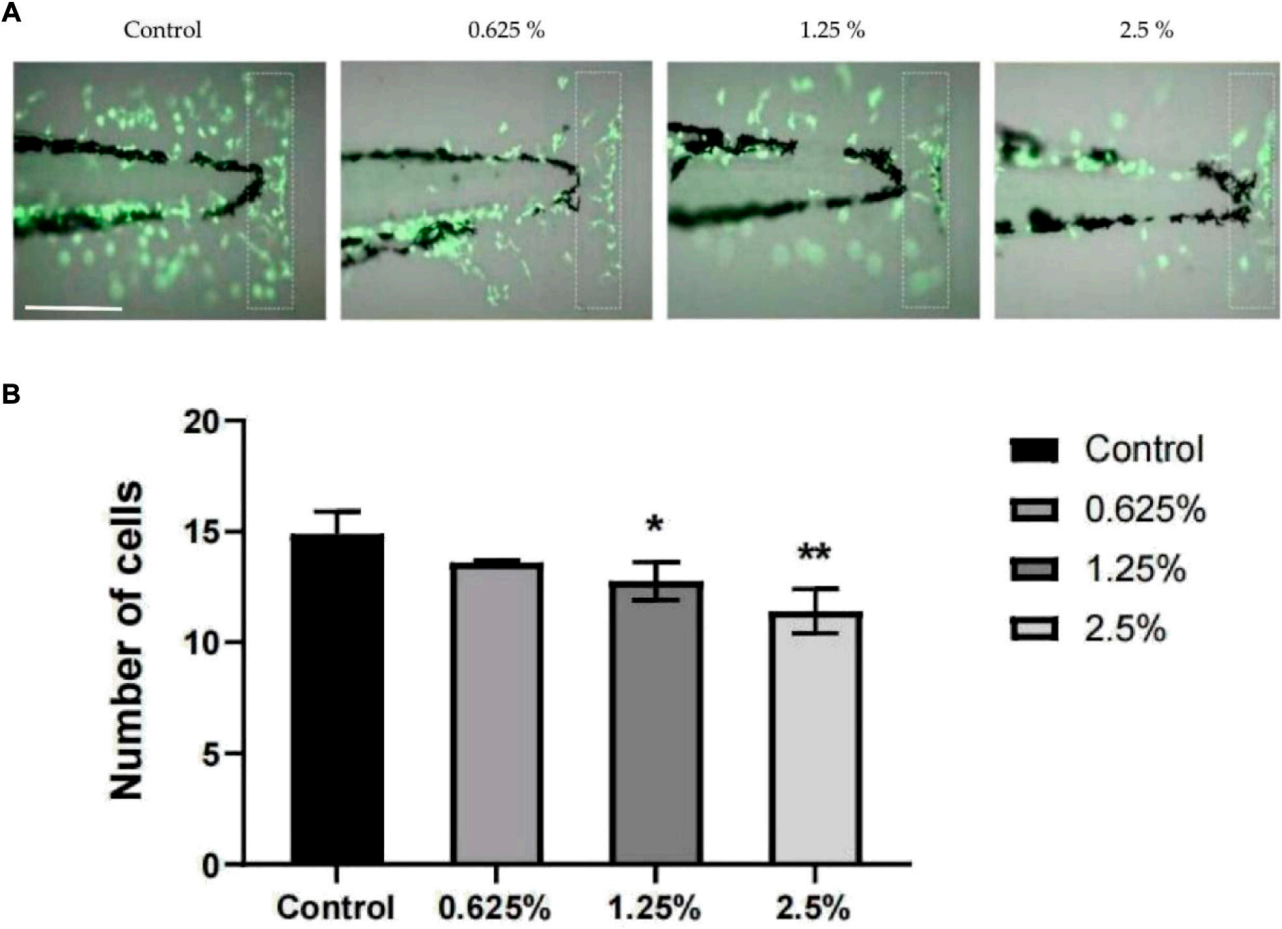
Anti-inflammatory effect of vine tea fermented broth, the scale in the figure is 200 μm. **(A)** Distribution of inflammatory cells in the tail of zebrafish larvae induced by tail cutting. **(B)** Number of cells recruited in the caudal (*p < 0.05, **p < 0.01 vs. Control group).

The zebrafish tail docking inflammation model is a traumatic inflammation model. The tail docking treatment can induce local damage to the zebrafish tails and promote the immune response of immune cells (Elks et al., 2011). With an increase in the concentration of vine tea fermentation broth, the aggregation of inflammatory cells was gradually inhibited (Figure 5A). The number of inflammatory cells recruited in the caudal fin of a single zebrafish at 6 h gradually decreased with the rise of the concentration of the vine tea fermentation broth (Figure 5B). The 1.25% group was significantly different (*p* < 0.05) and the 2.5% group was more significantly different (*p* < 0.01) compared with the control group. The zebrafish tail docking inflammation model proves the vine tea fermented broth has a certain anti-inflammatory ability. The polyphenolic compounds in the fermented vine tea have an important effect on the anti-inflammatory properties of the human body, avoiding the trouble of inflammation (Pudziuvelyte et al., 2020).

### 3.7 Eye irritation/corrosion of vine tea fermentation broth

The 0.625, 1.25 and 2.5% vine tea fermentation broths respectively after contacting the chorioallantoic membrane (CAM) for 3 min and the score of positive control (0.1 mol/L NaOH, Texapon ASV) on CAM are shown in Supplementary Material. The results show that only the 2.5% group showed a small amount of vascular hemorrhage, without vascular dissolution or coagulation. The 0.625%–2.5% vine tea fermented broths were judged as no/light irritation, which demonstrates the safety of the vine tea fermented broths.

## 4 Conclusion

A vine tea fermentation broth was developed by microbial fermentation and enzyme degradation technology, and its feasibility as a functional food was discussed, which provided a new idea for a healthy diet. This study screened various probiotics to obtain the most suitable strain for the vine tea fermentation, optimized the fermentation conditions, and degraded it with a new cellulase Cel 906. Compared with previous reports, this method extracted more flavonoids, polyphenols, and polysaccharides from vine tea. Microbial fermentation and cellulase degradation is key to the enrichment and precipitation of effective substances. To explore the characteristic of this vine tea fermentation broth, we conducted *in vitro* antioxidant experiments and found its strong scavenging ability against DPPH, ABTS, •OH, O2•− free radicals. When the fermented broth of vine tea was used on mouse melanoma B16-F10 cells. It inhibited the activity of tumor cells, the activity of intracellular tyrosinase, and the synthesis of melanin. Then the anti-inflammatory experiment with the zebrafish tail docking in-flammation model found the vine tea fermented broth inhibited the production of in-flammatory cells. The chicken embryo chorioallantoic membrane test verified that the vine tea fermentation broth within the effective concentration range is not irritating or corrosive. The fermented broth of vine tea is a kind of food with functional substances *in vitro* and is rich in bioactive substances that are beneficial to human health. Further experimental and clinical studies can be carried out to confirm the application of this vine tea fermentation broth in the food industry.

## Data Availability

The original contributions presented in the study are included in the article/Supplementary Material, further inquiries can be directed to the corresponding authors.

## Nomenclature

### Abbreviations

YPG: yeast-extract-powder peptone glucose
MRS: man-rogosa-sharpe
DMSO: Dimethyl sulfoxide
RPMI-1640: roswell Park Memorial Institutefoetal-1640
α-MSH: α-melanocyte-stimulating hormone
FBS: fatal bovine serum
MTT: 3-(4,5-dimethylthiazol-2-yl)-2,5-diphenyltetrazolium bromide
DPPH: 1,1-Diphenyl-2-trinitrophenylhydrazine
TRIS: tris(hydroxymethyl)aminomethane
Fe^2+^-EDTA: ferrous ethylenediaminetetraacetic acid
ABTS: 2,2′amino-bis (3-ethyl-benzothiazolinesulfonic acid-6) ammonium salt
HET-CAM: hens egg test-chorioallantoic membrane
Texapon ASV: sodium magnesium laury-myristyl-6- ethoxy-sulphate
ES: endpoint score.
