# Supplementary material for: Effects of novel cellulase (Cel 906) and probiotic yeast fermentation on antioxidant and anti-inflammatory activities of vine tea (Ampelopsis grossedentata)
Source: Front Bioeng Biotechnol. 2022 Sep 15;10:1006316. doi: 10.3389/fbioe.2022.1006316 (PMC9521311; doi:10.3389/fbioe.2022.1006316)
Supplement: Supplementary file 1 [file DataSheet1.docx]

Supplementary Material

**Supplementary Table 1.** Endpoint scoring result evaluation

| **Endpoint Score (ES)** | **Stimulus Classification** |
| --- | --- |
| ES≤12 | No/light irritation |
| 12<ES<16 | Moderate irritation |
| ES≥16 | Strong irritation /corrosion |

**Supplementary Table 2.** Level range of Plackett-Burman design

| **Number** | **Factor** | **Unit** | **Low Level** | **High Level** |
| --- | --- | --- | --- | --- |
| A | Fermentation Time | h | 72 | 120 |
| B | Temperature | ºC | 34 | 36 |
| C | Inoculation of Strains | % | 1 | 5 |
| D | Concentration | g/L | 66.7 | 200 |
| E | Glucose Addition | % | 1 | 3 |
| F | Urea Addition | % | 0.2 | 0.4 |
| G | pH |  | 7 | 9 |

**Supplementary Table 3.** Plackett-Burman design and test results

| **Run** | **A** | **B** | **C** | **D** | **E** | **F** | **G** | **Clearance Rate (%)** |
| --- | --- | --- | --- | --- | --- | --- | --- | --- |
| 1 | 1 | 1 | 1 | -1 | -1 | -1 | 1 | 68.5 |
| 2 | 1 | -1 | 1 | 1 | 1 | -1 | -1 | 78.99 |
| 3 | -1 | -1 | 1 | -1 | 1 | 1 | -1 | 41.55 |
| 4 | -1 | -1 | -1 | -1 | -1 | -1 | -1 | 43.23 |
| 5 | 1 | -1 | -1 | -1 | 1 | -1 | 1 | 53.78 |
| 6 | -1 | 1 | 1 | 1 | -1 | -1 | -1 | 66.28 |
| 7 | -1 | 1 | -1 | 1 | 1 | -1 | 1 | 61.76 |
| 8 | -1 | 1 | 1 | -1 | 1 | 1 | 1 | 40.93 |
| 9 | -1 | -1 | -1 | 1 | -1 | 1 | 1 | 54.56 |
| 10 | 1 | 1 | -1 | 1 | 1 | 1 | -1 | 90.17 |
| 11 | 1 | -1 | 1 | 1 | -1 | 1 | 1 | 75.19 |
| 12 | 1 | 1 | -1 | -1 | -1 | 1 | -1 | 64.07 |

**Supplementary Table 4.** Plackett-Burman analysis of variance

| **Source** | **Sum of Squares** | **df** | **Mean Square** | **F-Value** | **p-value (Prob>F)** | **Saliency List** |
| --- | --- | --- | --- | --- | --- | --- |
| Model | 2583.2 | 7 | 369.03 | 23.24 | 0.0044 | significant |
| A | 1244.81 | 1 | 1244.81 | 78.4 | 0.0009 | 1 |
| B | 163.1 | 1 | 163.1 | 10.27 | 0.0327 | 3 |
| C | 1.36 | 1 | 1.36 | 0.086 | 0.7843 | 7 |
| D | 1096.72 | 1 | 1096.72 | 69.08 | 0.0011 | 2 |
| E | 1.94 | 1 | 1.94 | 0.12 | 0.7445 | 6 |
| F | 3.24 | 1 | 3.24 | 0.2 | 0.6746 | 5 |
| G | 72.03 | 1 | 72.03 | 4.54 | 0.1002 | 4 |
| Residual | 63.51 | 4 | 15.88 |  |  |  |
| Cor Total | 2646.71 | 11 |  |  |  |  |

**Supplementary Table 5.** Level range of Box-Behnken design

| **Number** | **Factor** | **-1** | **0** | **1** |
| --- | --- | --- | --- | --- |
| A | Fermentation Time (h) | 72 | 96 | 120 |
| B | Concentration (g/L) | 66.7 | 133.35 | 200 |
| C | Temperature (ºC) | 34 | 35 | 36 |

**Supplementary Table 6.** Box-Behnken design and results

| **Run** | **A: Fermentation Time** | **B: Concentration** | **C: Temperature** | **Clearance Rate (%)** |
| --- | --- | --- | --- | --- |
| 1 | 96 | 200 | 34 | 59.15 |
| 2 | 96 | 66.7 | 34 | 76.1 |
| 3 | 120 | 66.7 | 35 | 63.9 |
| 4 | 96 | 200 | 36 | 57.2 |
| 5 | 96 | 66.7 | 36 | 55.15 |
| 6 | 120 | 133.35 | 34 | 52.06 |
| 7 | 96 | 133.35 | 35 | 86.01 |
| 8 | 96 | 133.35 | 35 | 84.23 |
| 9 | 72 | 133.35 | 34 | 51.2 |
| 10 | 120 | 200 | 35 | 72.11 |
| 11 | 96 | 133.35 | 35 | 84.09 |
| 12 | 72 | 66.7 | 35 | 77.94 |
| 13 | 72 | 133.35 | 36 | 60.1 |
| 14 | 72 | 200 | 35 | 64.1 |
| 15 | 120 | 133.35 | 36 | 69.1 |
| 16 | 96 | 133.35 | 35 | 83.91 |
| 17 | 96 | 133.35 | 35 | 84.07 |

**Supplementary Table 7.** Response Surface analysis of variance

| **Source** | **Sum of Squares** | **Degree of freedom** | **Mean Square** | **F-Value** | **p-value (Prob>F)** | **Significance** |
| --- | --- | --- | --- | --- | --- | --- |
| Model | 2152.79 | 9 | 219.2 | 74.87 | 0.0243 | * |
| A-Fermentation Time | 1.83 | 1 | 1.83 | 0.037 | 0.8522 |  |
| B-Concentration | 52.69 | 1 | 52.69 | 1.07 | 0.3346 |  |
| C-Temperature | 1.16 | 1 | 1.16 | 0.024 | 0.8824 |  |
| AB | 121.55 | 1 | 121.55 | 2.48 | 0.1595 |  |
| AC | 16.56 | 1 | 16.56 | 0.34 | 0.5795 |  |
| BC | 90.25 | 1 | 90.25 | 1.84 | 0.2172 |  |
| A^2 | 369.45 | 1 | 369.45 | 7.53 | 0.0288 | * |
| B^2 | 131.21 | 1 | 131.21 | 2.67 | 0.146 |  |
| C^2 | 1213.94 | 1 | 1213.94 | 24.74 | 0.0016 | ** |
| Residual | 343.52 | 7 | 49.07 |  |  |  |
| Lack of Fit | 340.47 | 3 | 153.49 | 148.99 | 0.0752 |  |
| Pure Error | 3.05 | 4 | 0.76 |  |  |  |
| Cor Total | 2496.31 | 16 |  |  |  |  |

*p<0.05, **p<0.01

**Supplementary Table 8.** Positive control scores for chicken embryo experiments.

| **Positive Control** | **Vascular Changes** | **Endpoint score** |
| --- | --- | --- |
| 0.2 % NaOH | Mild coagulation | 1 |
| 0.3 % NaOH | Moderate coagulation | 2 |
| 0.5 % NaOH | Severe coagulation | 3 |
| 0.5 % Texapon ASV | Mild bleeding; Mild vascular melting | 1; 1 |
| 1 % Texapon ASV | Moderate bleeding; Moderate vasolysis | 2; 2 |
| 5 % Texapon ASV | Severe bleeding; Severe vasolysis | 3; 3 |

**Supplementary Table 9.** Endpoint scores and irritation classification

| **Samples** | **Endpoint score** | **Irritation Classification** |
| --- | --- | --- |
| Negative control | 0 | Non-irritating |
| 0.625% group | 0 | No/light irritation |
| 1.25% group | 0 | No/light irritation |
| 2.5% group | 0.67 | No/light irritation |

**
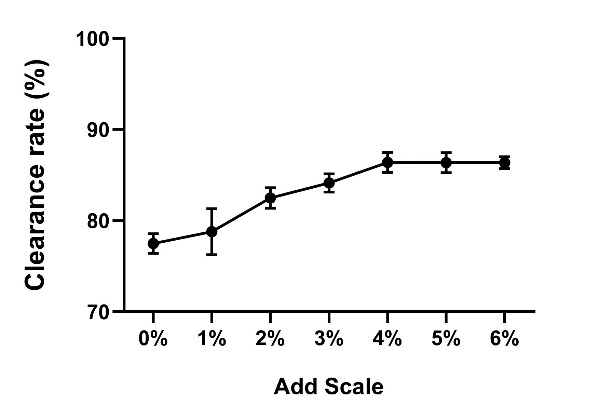
**

**Supplementary Figure 1.** The effect of the addition amount of Cel 906 on the antioxidant activity of fermentation broth. (relative to the volume of fermentation broth)


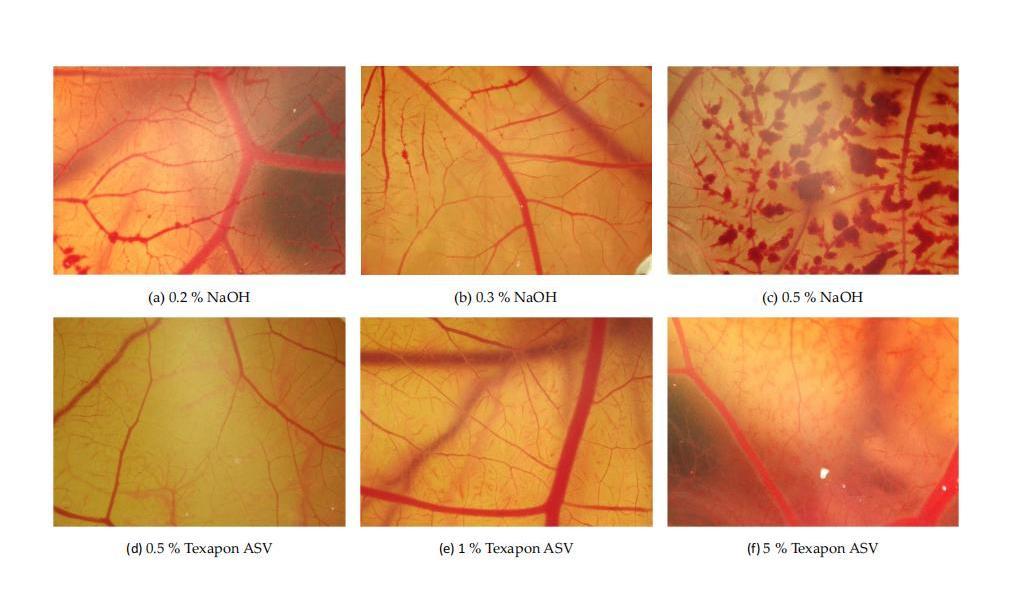


**Supplementary Figure 2.** Vascular changes after CAM exposure to positive control for 3 min.


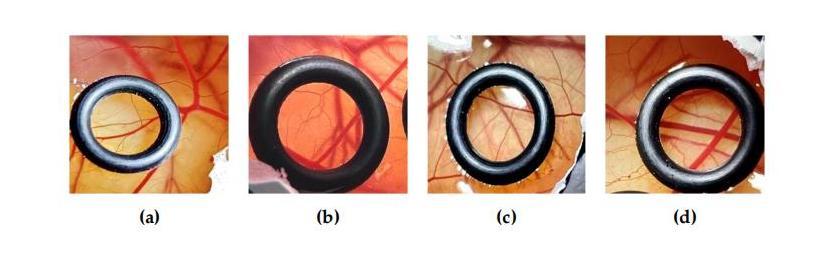


**Supplementary Figure 3.** CAM after exposure to the reagent for 3 min. (a) control; (b) 0.625%; (c) 1.25%; (d) 2.5% (the black Teflon ring showing the experimental environment).
